# Autonomous detection and sorting of litter using deep learning and soft robotic grippers

**DOI:** 10.3389/frobt.2022.1064853

**Published:** 2022-12-01

**Authors:** Elijah Almanzor, Nzebo Richard Anvo, Thomas George Thuruthel, Fumiya Iida

**Affiliations:** ^1^ The Bio-Inspired Robotics Lab, Department of Engineering, University of Cambridge, Cambridge, United Kingdom; ^2^ Costain Group PLC, Cambridge, United Kingdom

**Keywords:** AI-driven control, soft robotics, deep learning, visual servoing, litter picking

## Abstract

Road infrastructure is one of the most vital assets of any country. Keeping the road infrastructure clean and unpolluted is important for ensuring road safety and reducing environmental risk. However, roadside litter picking is an extremely laborious, expensive, monotonous and hazardous task. Automating the process would save taxpayers money and reduce the risk for road users and the maintenance crew. This work presents LitterBot, an autonomous robotic system capable of detecting, localizing and classifying common roadside litter. We use a learning-based object detection and segmentation algorithm trained on the TACO dataset for identifying and classifying garbage. We develop a robust modular manipulation framework by using soft robotic grippers and a real-time visual-servoing strategy. This enables the manipulator to pick up objects of variable sizes and shapes even in dynamic environments. The robot achieves greater than 80% classified picking and binning success rates for all experiments; which was validated on a wide variety of test litter objects in static single and cluttered configurations and with dynamically moving test objects. Our results showcase how a deep model trained on an online dataset can be deployed in real-world applications with high accuracy by the appropriate design of a control framework around it.

## 1 Introduction

**FIGURE 1:**
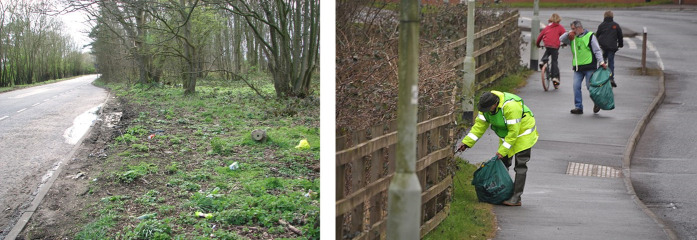
The left image shows the roadside pollution typically observed in the UK [taken from Daily Mail Online (2021)]. Right image is an example of maintenance crews undertaking the laborious and dangerous litter picking task [taken from BBC News (2018)].

Roadside litter poses a severe safety and environmental risk for road users, wildlife and the maintenance crews who clean it up (National Highways, 2022) (see Figure 1). According to National Highways, which is responsible for maintaining and cleaning the United Kingdom’s strategic road network, taxpayers in the United Kingdom are paying £4.8 million per year for cleaning up roadside litter (Be Wiser, 2016).

Litter generally refers to any misplaced or solid waste. It appears in different formats including but not limited to sweet wrappers, drinking containers, fast food packaging, cigarette ends, small bags etc. Many countries around the world have seen an increase in roadside litter (Kaza et al., 2018; Karimi and Faghri, 2021; Smith, 2022). Several sources of litter on the roads were identified, including pedestrians, motorists, household trash, and others as reported (Karimi and Faghri, 2021). Overtime build-up of litter alongside the road has increased due to population growth, and an increase in the road network and users. The problem has been accelerated with the global pandemic COVID-19 with personal protective equipment (KAB, 2020). The US spend nearly 11.5 billion dollars each year for removing all sorts of litter (Karimi and Faghri, 2021). Depending on the amount of litter, it can pose safety hazards and can cause operational issues for road users (National Highways, 2022). These items can interrupt the traffic flow and cause delays. More importantly, such a situation can directly or indirectly lead to a collision. In the US, road debris-related crashes contributed to approximately 50,658 of the police-reported cases (Tefft, 2016). The environmental impact of roadside litter goes far beyond the boundaries of the roadway network. The toxic materials contained in such garbage can be washed away with rain and contaminate the soil and groundwater (Kurmus and Mohajerani, 2020). Furthermore, some of the litter end up in streams, rivers, drains and ocean. A study has shown that plastics decompose into microplastics over time in the ocean and can end up in the food chain (Thiele et al., 2021). A number of approaches have been proposed to reduce or prevent litter. From a societal perspective, there are three major approaches to litter prevention; education and public awareness, receptacles’ design, and consequence control (Prata et al., 2019).

There are two key challenges with garbage disposal. First, the item has to be collected from the disposed location and then sorted for its appropriate recycling process. Traditional garbage cleaning is performed by paid workers, organisations, volunteers and charities sent on-site to pick up items alongside the road. Manual picking is a tedious, boring and repetitive task (Deery et al., 2019). Workers spend most of their time alongside roads with motorists, posing a safety risk. Furthermore, hazardous material associated with litter exposure can lead to diseases and infections. A review on roadside litter paper estimates that 8.3 billion tons of plastic have been created over the past 50 years, and only 9% have been recycled, indicating a large scope for improvement in the sorting process (Karimi and Faghri, 2021).

To automate the cleaning process and improve the safety of workers, litter detection algorithms and robotic systems have been developed over the past few years. Some of the first few works use novel sensing technologies for waste identification. For example, an automatic trash detection algorithm using an ultrasonic sensor was proposed in (Kulkarni and Junghare, 2013). A sorting solution for mixed recycled aggregates using near-infrared technology was proposed in (Vegas et al., 2015). A multi-material classification technique based on the utilisation of thermal imaging for sorting dry recyclables from municipal solid waste was proposed in (Gundupalli et al., 2017). A laboratory test was conducted on four broad categories of dry recyclables and obtained a classification success rate in the range of 85–96%. Due to recent advancements in deep learning algorithms, object detection using visual data has become more popular. For example, a deep learning-based pavement inspection framework for detecting and localising pavement defects simultaneously with garbage detection has been reported in (Ramalingam et al., 2021).

One of the first implementations of a robotic device for litter picking, the ZenRobotics recycler robotic system, uses machine learning and a robotic manipulator to pick recyclable objects from a conveyor belt (Lukka et al., 2014). A 3D high-resolution sensor is used to get an isometric 2D map of the conveyor, then a learning-based method is used for object recognition and manipulation. A similar approach using a fast parallel manipulator with a suction gripper, for sorting items on a conveyor was investigated in (Raptopoulos et al., 2020). However, these works have not been tested in real-world scenarios. Bai et al. (2018) presented a novel garbage pickup robot tested on grass using a learning-based object segmentation algorithm. Liu et al. (2021) developed a comprehensive system that uses deep learning for object segmentation and classification of different classes. Incorporated with a mobile robot, a grasp localization method to identify a suitable grasp pose to pick the garbage from the ground was also developed.

This paper proposes a cost-effective strategy for litter picking using a robotic manipulator. Our contributions are as follows:

• A modular approach to robotic development to minimise costs and development time. The robot is comprised of inexpensive or off-the-shelf components which are improvable over time.
• Distinct from other works, we simplify and improve the robustness of our manipulation system by using soft robotic grippers and a real-time visual-servoing controller, requiring only a 2D colour camera for picking and binning objects of variable sizes and shapes even in dynamic environments.
• We use a learning-based object detection and segmentation algorithm trained on the online TACO dataset for identifying and classifying garbage, making our framework easily transferable to additional objects and classes by simply retraining the object detection network.

Our results indicate a high grasp success rate and good recycling accuracy.

The rest of the paper is organized as follows. Section 2 presents an overview of the proposed robot system, including hardware configuration and control architecture. The approaches our methodology employ to realize the litter-picking including perception, object tracking and experimental results, which demonstrates the proposed litter-picking robot’s effectiveness, are given in Section 3. Finally, the discussion and conclusions are in Section 4.

## 2 Methodology

### 2.1 Roadside litter picking robotic system

**FIGURE 2:**
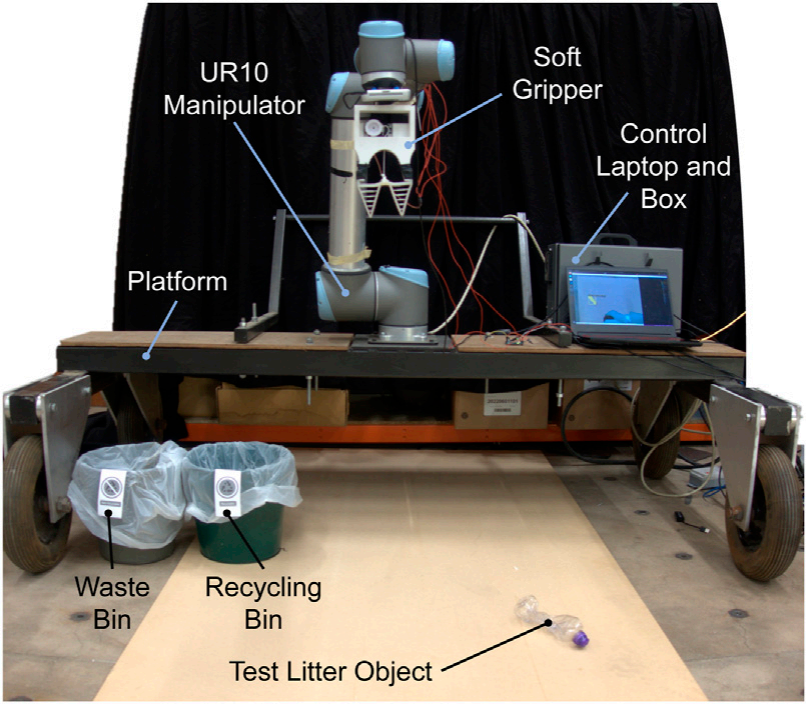
The LitterBot and its components.

The roadside litter-picking robot (LitterBot) is shown in Figure 2. To minimise the development time and cost of the prototype robot, inexpensive components were deliberately chosen and integrated in a modular manner. The robot comprises a UR10 6 Degree-of-Freedom (DoF) robotic manipulator with a Fin Ray type soft end-effector. The end-effector mounts a RealSense D415 colour and depth camera for the machine vision with a 1920 × 1080 resolution. The depth information, however, is not used. The UR10 manipulator is mounted on a wheeled platform that is 2 m wide, 55 cm high and 140 cm long. Note that the wheeled platform is kept stationary, however, it can easily be towed by a vehicle for movement. The robot has a semi-circle working area with a radius of 1.3*m*. The Robotics Operating System (ROS) is employed for the software architecture running on a control laptop. Control of the manipulator is done through the built-in motion and kinematic controllers.

### 2.2 Soft gripper

**FIGURE 3:**
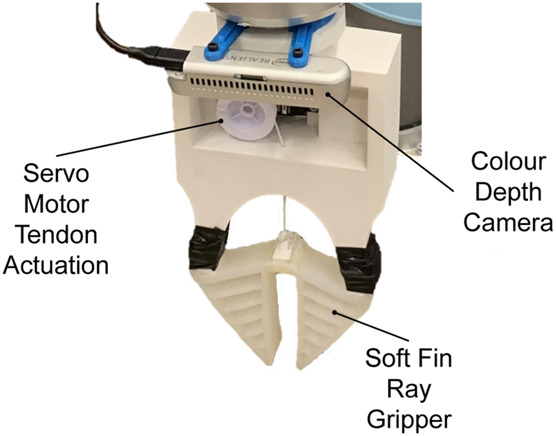
The soft Fin Ray type gripper with the depth camera.

The soft gripper employs two Fin Ray fingers driven by a single servo motor. These structures have several advantages compared to other soft grippers such as ease of use, minimal actuation and the capability to grasp a wide variety of objects (Crooks et al., 2016). The “V” shape structure with layers of crossbeams at the centre allows for the mechanically passive adaption to the geometry of the object applying the force. Unlike, other typically used soft grippers like PneuNets (Mosadegh et al., 2014) or Universal Grippers (Brown et al., 2010; Sakuma et al., 2018), Fin Rays also do not have the risk of failure when punctured. The gripper is given in Figure 3.

The material used for the Fin Ray fingers is the Dragon Skin 30 silicone. The mould for casting was 3D printed. The fingers are attached to the 3D-printed PLA gripper base (see Figure 3) which is mounted to the UR10 robot. A tendon attached to the bottom of the fingers is tethered to the pulley of a servomotor for actuation. Control of the gripper is achieved using an Arduino microcontroller.

### 2.3 Visuo-motor control architecture

#### 2.3.1 Detectron 2 for object segmentation and classification

The vision system uses the Detectron 2 version of Mask Region Convolutional Neural Network (Mask R-CNN) for the litter instance segmentation and classification. This outputs both masks and bounding boxes. To minimise the developmental cost of the modular integration, we deploy the Resnet50 backbone due to its trade-off between high average precision performance and inference time on the COCO dataset compared to other pre-trained network weights available (Facebook Research, 2021). The network was implemented in Python 3 using PyTorch.

Principal component analysis (PCA) is used on the masks to determine the angular orientation of the objects. The principal axis corresponds to the long-ways orientation of the target mask. The variance matrix *A* of the segmented grey-scale mask data is used to find the first eigenvalue *λ*. This is used to solve for the eigenvector **v**. The angle of the target object is obtained using inverse tan on the eigenvector. The angle is constrained between − 90 and 90°.

$$\left(A-\lambda I\right)=v$$ (1)

$$\theta =\tan^{-1}\left(\frac{v_{2}}{v_{1}}\right)$$ (2)

**FIGURE 4:**
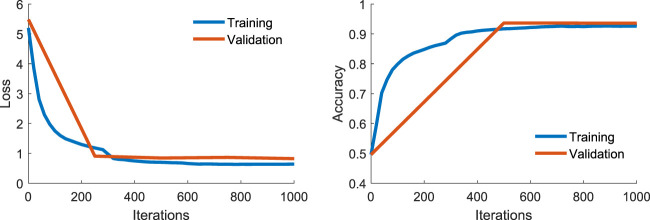
Training and validation loss (left) and accuracy (right) for the TACO dataset.

The Trash Annotations in Context (TACO) dataset was used to train the network (Proença and Simões, 2020). It consists of 1500 images, with 60 classes. TACO was divided into a 90% and 10% train and validation set respectively. The network was trained on Google Colab using a batch size of 512 for 1000 iterations (approximately 350 epochs). The training and validation loss is given in Figure 4. The images were automatically resized to the default size of 800 × 800. Overfitting occurs at around 300 to 500 iterations, hence the ultimate network weights were taken at 500 iterations. The network achieves a 94% accuracy on the validation set. Deployment inference and evaluation were done using the control laptop’s graphical processing unit (RTX 2060m).

#### 2.3.2 Visual servoing-based litter picking

**FIGURE 5:**
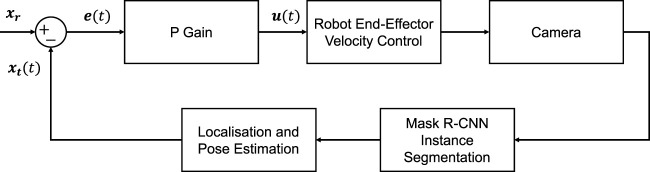
Visual servo control block.

The robot employs a velocity-based eye-in-hand visual-servoing scheme for litter picking (see Figure 5). Given a target object present in the camera view located at pixel coordinates **x**_**t**_ = (*x*_*p*_, *y*_*p*_), the pixel error **e** = [*e*_*x*_, *e*_*y*_] to the reference pixel coordinates **x**_**r**_ = (*x*_*r*_, *y*_*r*_), corresponding to the centre of the gripper, is multiplied by a proportional gain *K*_*p*_. This forms the end-effector Cartesian velocity control input to the robot, given in Eq. 3.

$$U\left(t\right)=K_{p}e\left(t\right)$$ (3)

A value of 0.0005 was used for *K*_*p*_. Both *K*_*p*_ and the reference pixel coordinates were found empirically. Pixel target coordinates (*x*_*p*_, *y*_*p*_) are taken as the centre of the target’s Detectron2 bounding box. The robot picks the target based on the detected object with the largest mask. Detectron2 has a frequency of 7Hz, hence a low proportional gain is implemented to retain control stability. The control input *U*(*t*) is given to the UR10 in-built function *speedl* to achieve the closed-loop control. Once the error is sufficiently small, the visual-servoing process is terminated. **e** < 2 was empirically found to give reasonable grasping accuracy. The robot then proceeds to grab the object. For moving to the object, the final current *X* and *Y* Cartesian end-effector positions are taken, however, the Z position is assumed to be fixed and estimated beforehand. The angle estimated by PCA is then used to re-orient the gripper as it drops such that the thinnest width of the object corresponds to the mouth of the fin ray gripper.

## 3 Litter picking experiments and results

### 3.1 Experimental protocol

**FIGURE 6:**
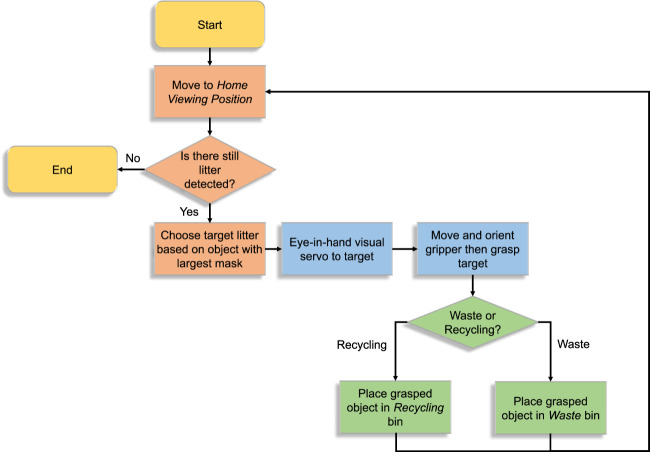
Picking and binning process.

Three litter-picking experiments were conducted. For all three, the test objects are laid on the ground. The aim of the experiments was to validate the holistic performance of the robot when *picking and binning* litter. This process is broken down into three distinct steps (see the flowchart in Figure 6); litter identification (orange blocks), grasping (blue blocks), and classified binning (green blocks).

The robot first goes into a home viewing position, with a camera view that is parallel to the ground. The current litter target is chosen as the identified object with the largest segmentation mask. The robot then grasps the target object based on the eye-in-hand visual servoing method. The robot then drops the grasped litter into either the “Waste” or “Recycling” bin depending on the predicted class of the target. The 60 classes in the TACO dataset were manually divided into “Waste” or “Recycling” categories depending on the materials they are comprised of. This was implemented in the software using a rule-based approach, for example, objects identified as “drink can” are classified as recycling, and objects identified as “other plastic wrapper” which contain non-recyclable materials are classified as “waste”. Although the dataset has 60 possible classes, only four object types were used in the experiments. The classes considered as “Recycling” are “Clear plastic bottle” and “Drink can”. The classes considered as “Waste” are “Other plastic wrapper” and “Plastic film”. We believe these are the most prominent types of pollution found in urban areas. More importantly, these objects have not been seen in the training data.

### 3.2 Static individual object litter picking

**FIGURE 7:**
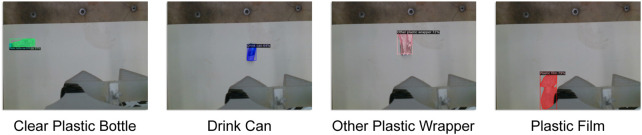
Example initial views for the four object types before the *pick and bin* process is executed.

**FIGURE 8:**
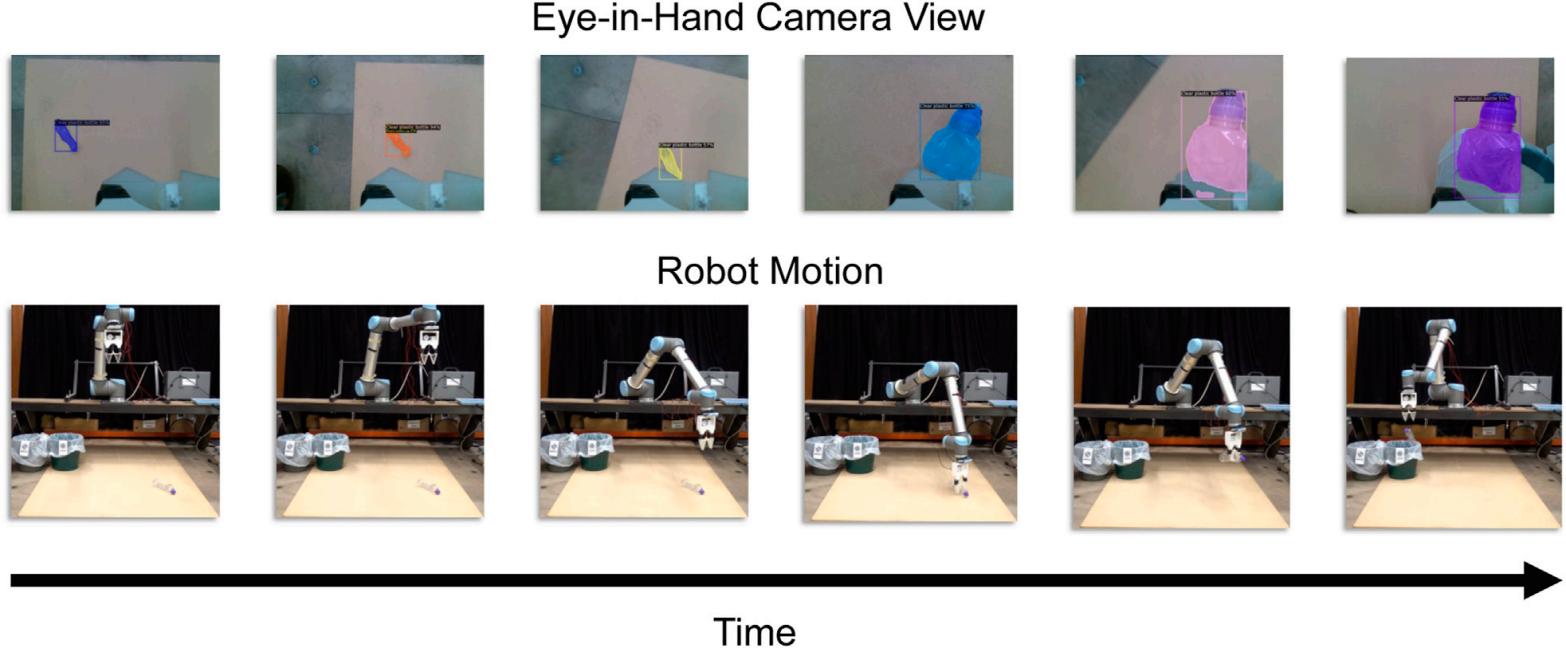
An example of the Visual Servo graph for dynamic obstacles.

In this first experiment, the four objects were *picked and binned* individually. For each object, ten trials were undertaken. At each trial, the object was placed in a random pose on the floor in front of the LitterBot (see Figure 7). A single *pick and bin* process is considered a success if the robot could detect, grasp and dump the object in the correct bin. An example of the visual-servoing is given in Figure 8.

From Table 1, it can be seen that the robot is able to correctly *pick and bin* a wide variety of objects with success rates of at least 80%. None of the failures was from object misclassification suggesting a relatively robust vision network when detecting a single object even with such a small dataset. One reason for unsuccessful attempts was due to the object being dropped by the gripper as a result of non-robust grasps. A mechanically stiffer gripper, however, should circumvent this issue. Another reason for unsuccessful attempts were objects being placed outside of the robot’s workspace. This can be easily addressed by placing the manipulator on a mobile platform. The time it takes to pick the objects is highly dependent on where the object is placed relative to the robot. This was the reason for the relatively high standard deviations. The robot, however, is able to successfully pick up the objects it has previously dropped upon further attempts.

**TABLE 1:** Success rates and average times for the ten individual *picking and binning* tests for the four objects.

Test object	Success rate (%)	Mean time (*s*)
*Recyclable*		
Clear Plastic Bottle	80	22.3 ± 5.8
Drink Can	80	19.8 ± 6.9
*Waste*		
Other Plastic Wrapper	90	22.1 ± 11.1
Plastic Film	90	25.5 ± 10.9

### 3.3 Dynamic object litter picking

There are many factors which can contribute to a dynamic roadside environment. These factors include moving litter and garbage as a result of wind or inclined surfaces, the robot itself moving within the environment, environmental and weather conditions, and cars and pedestrians. However, to test the efficacy of our framework, specifically the performance of the visual servoing approach combined with the gripper and the vision network, we have chosen to address only the dynamically moving garbage and litter. Here, a clear plastic bottle was dynamically moved around the workspace to simulate low-speed wind. A string was tied around the neck of the bottle, which was used to manually pull the bottle in random directions for a random time period, after which the movement is stopped. This assumption holds for litter that has settled down on the side roads, typically seen in roadside pollution. Note that this experiment is likely untrue for other litter such as lightweight shopping plastic bags. Five trials were undertaken.

**FIGURE 9:**
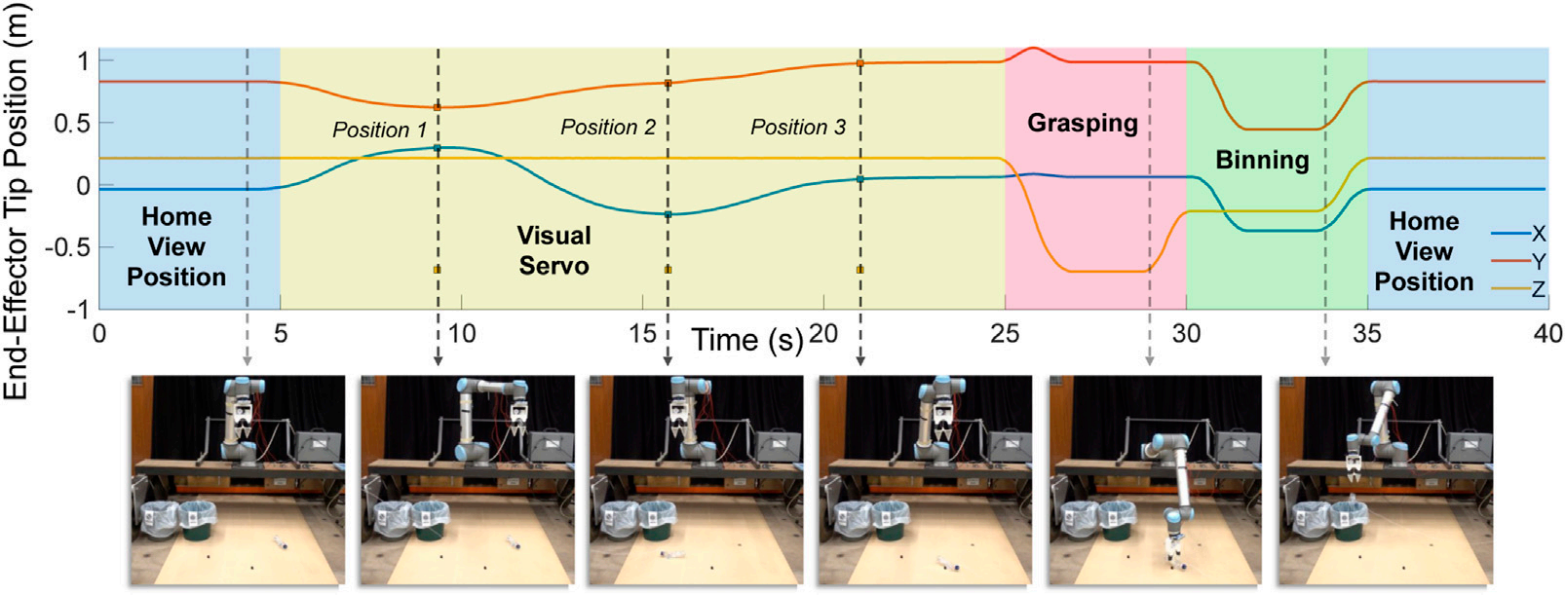
The left image shows the roadside pollution typically observed in the UK [taken from Daily Mail Online (2021)]. Right image is an example of maintenance crews undertaking the laborious and dangerous litter picking task [taken from BBC News (2018)].

An example of dynamic visual-servo tracking is given in Figure 9. In this example, the bottle was moved between three different positions. The yellow region of the tip position graph shows the ability of the robot to follow the dynamic object.

From Table 2, it can be seen that the visual-servoing approach achieves reasonable performance in tracking moving litter for *picking and binning* in all five trials. The time it takes for the robot to *pick and bin* the object is also dependent on how long the object has moved until it has settled to a fixed location.

**TABLE 2:** *Pick and Bin* success and process execution time for the five trials with the dynamic object.

Clear plastic bottle	*Pick and bin* success	Time (*s*)
Trial 1	*Success*	67.4
Trial 2	*Success*	20.3
Trial 3	*Success*	28.8
Trial 4	*Success*	75.3
Trial 5	*Success*	30.9
*Mean*		44.6 ± 24.9

### 3.4 Cluttered objects litter picking

**FIGURE 10:**
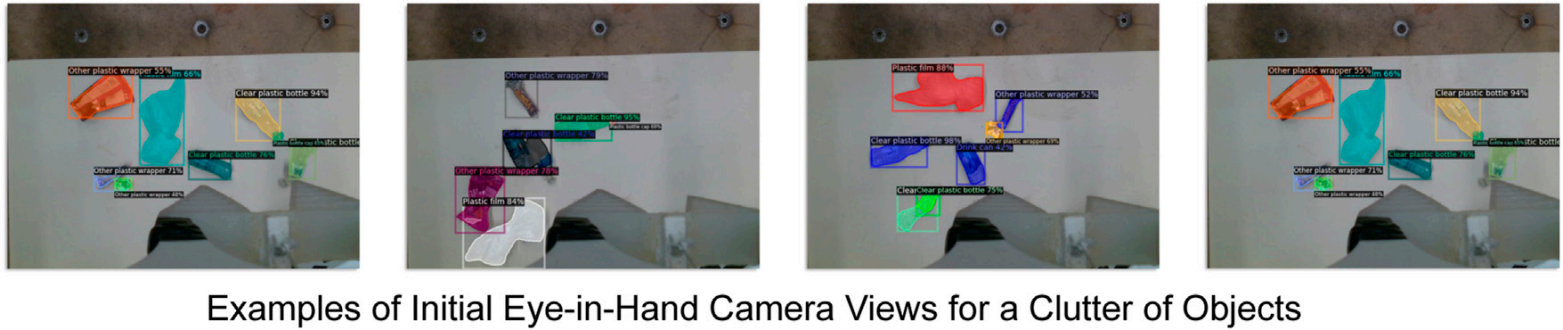
Example initial views for a clutter of objects before the *pick and bin* process is executed.

Finally, this experiment tests the performance of the robot’s *pick and bin* process when there are numerous cluttered objects. This simulates the cluttered conditions the LitterBot will likely encounter in a real-world deployment. Here, six test objects that fit the four classes are placed in random poses within the LitterBot’s working space (see Figure 10). Three of the objects were in categories that can be recycled, and the remaining three were waste. Nine trials were conducted. An additional plastic wrapper (of a chocolate bar) and a plastic water bottle were added. The objects used vary widely in size, geometry and material which is representative of typical roadside pollution (see Figure 1). The robot picks the current target depending on the object with the largest detected mask. Here, the soft gripper’s grasping success rate and litter binning classification were tested. The latter implicitly test the performance of the trained network when there are multiple objects within the scene.

From Table 3 (and Table 1 from the single static object experiment in Section 3.2), it can be seen that the gripper’s compliance and adaptability performs well for grasping the various objects. Only one object was dropped in all nine trials. Overall, the LitterBot achieves relatively high binning classification success rates for either waste or recycle binning. Relative to the first experiment, however, the vision network was more likely to misclassify litter objects (such as putting recyclable objects in the waste bin and *vice versa*) when the view is cluttered. This suggests a larger dataset might be required for real-world deployment.

**TABLE 3:** *Pick and Bin* grasping and litter classification success rates for the clutter of objects.

*Success rates %*			
Trial	*Grasping*	Waste binning	Recycle binning
Trial 1	100	100	0
Trial 2	100	100	100
Trial 3	100	100	100
Trial 4	100	66.7	66.7
Trial 5	100	66.7	100
Trial 6	83.3	33.3	100
Trial 7	100	100	66.7
Trial 8	100	100	100
Trial 9	100	100	100
*Mean*	98.1 ± 5.2	85.2 ± 22.8	81.5 ± 31.9

## 4 Discussion and conclusion

In this paper, we introduce the LitterBot, a roadside litter-picking robot prototype that is economically and computationally cost-effective. The robot uses the off-the-shelf Mask R-CNN (Detectron2) network for litter instance segmentation trained on the relatively small TACO dataset. Instance segmentation not only allows for the localisation of the detected objects within the image scene but also the inexpensive pose estimation using PCA. When augmented with 2D pixel real-time visual-servoing using the localised information and a soft-robotic gripper, the robot is highly successful in picking up and correctly binning a wide variety of objects which have drastically different weights, geometry, materials and recyclability. The robot’s success rate in *picking and binning* is consistently above or at least 80% for the various experiments. The use of an underactuated compliant and adaptable gripper allows for the robust grasping of arbitrarily shaped objects requiring minimal control. Pixel-based visual-servoing also has several advantages over open-loop control such as being less sensitive to frame transformation noise, and the ability to track dynamic objects even allowing for the re-picking of previously dropped objects.

The LitterBot also only requires 2D images for *picking and binning*, unlike prior works which require 3D point cloud images (Lukka et al., 2014; Raptopoulos et al., 2020; Liu et al., 2021) for planning appropriate grasp movements. Our method circumvents this through the use of a soft gripper which greatly simplifies the control complexity without sacrificing performance. This reduces economical costs as our soft gripper, although bespoke, is easy to fabricate and exponentially cheaper than off-the-shelf grippers. Point-cloud-based grasp planning also needs expensive depth cameras and additional grasp pose-detecting algorithms to tolerate noisy image depth data which would be prevalent in a real deployment. Our robot on the other hand only requires mask data and can easily be deployed on cheaper 2D cameras, making it more economically and computationally inexpensive. The robot is also easily scalable to additional objects by simply retraining the vision network on new 2D data which is far easier to obtain than point cloud-based data.

The deliberate use of modular components has the advantage of being improvable over time, which is easily extendable to more complex mechanisms and algorithms. The robot, however, is still a prototype and has scope for improvements before it is viable for real-world deployment. Although the gripper is adaptable, it is limited to objects that can fit within its grasp. One solution would be to incorporate an additional suction cup gripper such that larger objects such as pizza boxes can also be grasped. Future versions of the Fin Ray gripper will also include stiffer materials to increase the maximum graspable weight. A slight underperformance was also observed when multiple objects are present in the camera view. A larger dataset in the future will be beneficial for increasing the robustness of the vision system. Environmental factors such as variation in weather and lighting conditions as well as background and scenery will also be addressed in future work through more advanced and complex computer vision algorithms. Other networks such as YOLACT which was used in (Liu et al., 2021) will also be tested to increase the control frequency of the visual-servoing approach such that faster-moving objects can be tracked and objects can be grasped faster.

Further future work includes mounting the robot on a mobile robotic platform such that it can be autonomously deployed in the field. Two control schemes will also be considered, compared and evaluated in the future. The first is the “stop and bin” approach, which is already implemented in this work. The second, is where the robot can dynamically move whilst picking and binning. Algorithms for obstacle avoidance and picking order will also be developed in the future such that safety and energy efficiency can be additionally improved, as well as account for moving cars and pedestrians. Depth distance information will also be included in the servoing approach such that the robot can also grasp objects on inclines and non-planar surfaces. The improved robot will then be field-tested to fully test the efficacy of the LitterBot.

Overall, the simple yet robust and inexpensive control framework for the LitterBot performs well in cluttered and dynamic environments, thus showing promise for deploying autonomous systems for roadside litter-picking. This can greatly reduce roadside pollution as well as reduce costs, risks and hazards faced by users and maintenance crews.

## Data Availability

The raw data supporting the conclusions of this article will be made available by the authors, without undue reservation.
